# Student Self-Efficacy and Aptitude to Participate in Relation to Perceived Functioning and Achievement in Students in Secondary School With and Without Disabilities

**DOI:** 10.3389/fpsyg.2021.607329

**Published:** 2021-05-06

**Authors:** Karin Bertills, Mats Granlund, Lilly Augustine

**Affiliations:** ^1^School of Education and Communication, CHILD, SIDR, Jönköping University, Jönköping, Sweden; ^2^School of Health and Welfare, CHILD, SIDR, Jönköping University, Jönköping, Sweden

**Keywords:** self-efficacy, participation, functioning, achievement, disability, physical education, secondary school

## Abstract

School-based Physical Education (PE) is important, especially to students with disabilities whose participation in physical activities out of school is limited. The development over time of participation-related constructs in relation to students’ perceived functioning and achievement is explored. Students in mainstream inclusive secondary school self-rated their PE-specific self-efficacy, general school self-efficacy, aptitude to participate in PE, and perceived physical and socio-cognitive functional skills at two timepoints, year 7 and year 9. Results were compared between three groups of students with: disabilities (*n* = 28), high grades (*n* = 47), or low grades (*n* = 30) in PE. Over time, perceived physical skills of students with disabilities became strongly associated with self-efficacy and aptitude to participate. Perceived socio-cognitive skills in the study sample improved and had a positive effect on PE-specific self-efficacy. Efforts should be made to limit the accelerated negative impact of perceived restricted functioning of students with disabilities. Grading criteria need to be developed to comply with standards adapted to fit abilities of students with disabilities. Meaningful learning experiences appear to be created when participation is promoted and capacity beliefs (PE-specific self-efficacy) are boosted. Allocating resources to support the development of students’ socio-cognitive skills seem to have potential for overall positive school outcome.

## Introduction

Functional limitations in an educational setting may cause doubts about the self-efficacy and participation of students with disabilities. Self-perceptions of capacity and competence are aspects important for learning and whether a person makes an effort, practices skills, and persists on task until finished (Bandura, 1993). Self-efficacy beliefs predict learning outcomes and play an important role for the development, functioning, and achievement of adolescents (Bandura, 1993). Participating in physical activity is a fundamental right (UNESCO, 2015a), but owing to restricted functioning, the participation of persons with disabilities in extracurricular activities is limited (King et al., 2009). Compulsory secondary school in Sweden is mainstream, and inclusive school-based Physical Education (PE) is a context where *all* students must partake. School-based PE is therefore an important context for students with disabilities to share the benefits of physical activities with their typically functioning peers (Block and Obrusnikova, 2007; Bailey et al., 2009; Seymour et al., 2009; Grenier et al., 2014). The Swedish National Agency for Education (2018) curriculum states the equal value of all people, and the task of school is directed toward successful participation in society. However, irrespective of functional restrictions, grading criteria are the same for all students, and although support should be provided to students facing the risk of failing a subject, aide and assistance in PE is scarce (Morley et al., 2005; Tant and Watelain, 2016; Bertills et al., 2019). Apart from participatory gains in inclusive PE practices, self-efficacy beliefs may be boosted in PE by providing adolescents with an opportunity to acquire experiences and skills different to those achieved in typically academic school subjects (UNESCO, 2015b). Additional support, curricular adaptations, activity modifications, and individual accommodations may be needed to create meaningful learning experiences that students with disabilities will want to attend (Haegele and Sutherland, 2015). Developmental processes of self-efficacy (PE-specific and general), aptitude to participate, and perceived functioning are the focus of this study.

### Self-Efficacy Beliefs

Encountering new challenges, individuals consider whether they *can*, i.e., have the capability or not to succeed with the task. Self-efficacy refers to expectations of successful future performance in specific tasks (Bandura, 1997). Causal attribution, beliefs regarding the causes of events (Weiner, 1985), affects motivation, performance, and affective reactions mainly through beliefs of self-efficacy (Bandura, 1993). According to Bandura (1993), highly self-efficacious individuals invest energy into new tasks, choose more difficult tasks, practice persistently despite obstacles, and make great effort to finish the task. Failure is attributed to external factors, e.g., insufficient effort, making them more resilient to failure. Doubting their capacity, individuals with similar ability but with low self-efficacy have low aspirations of success, easily give up, assign failure to their lack of ability, and recover slowly, which gradually undermines their capability.

Self-efficacy beliefs differ from other self or expectancy constructs because they are task- and context-specific, aim for certain goals, and focus on the individuals’ own perceptions of capability. Beliefs in one’s capability may also spread across domains and influence choices made throughout life (Pajares and Urdan, 2006). Perceived self-efficacy beliefs include emotional, physical, mental, and social skills achieved by practice and interaction and the will to invest effort into engaging in the activity. The sources of self-efficacy beliefs are previous experiences of success, imitating role models, encouragement, and affective states (Bandura, 1994). Transitioning to adolescence in secondary school requires students to control their own learning more independently, and Zimmerman (2000) has shown that perceived self-efficacy, functioning, and learning are closely related. The predictive power of self-efficacy on academic achievement is well established in several subject areas (Pajares, 2003; Schunk, 2003; Gustafsson et al., 2010; Kitsantas et al., 2011). Empathetic self-efficacy predicts prosocial behaviors (Caprara et al., 2012), and students who initially report higher efficacy to self-regulate their actions later experience lower levels of problem behavior, achieve higher grades, and are more popular among their peers (Caprara et al., 2004). Less is known about how individuals’ self-efficacy is related to more practical school subjects, such as PE.

### Participation

Engagement with school is related to academic success, positive emotions and life satisfaction, and dis-engagement with negative school outcomes, such as ill-being (Upadyaya and Salmela-Aro, 2013). Students with disabilities participate less in physical recreational activities than their typically developing peers (King et al., 2009; Woodmansee et al., 2016). School-based PE offers opportunities for all students to participate in physical activity. However, the aptitude to participate in PE generally declines over time for all students (The Swedish Schools Inspectorate, 2018), unless students are involved in extracurricular physical activities (Säfvenbom et al., 2014). Participation is defined as “involvement in a life situation” in the International Classification of Functioning, Disability and Health for Children and Youth (ICF-CY) (World Health Organization, 2007). Attendance and involvement is the two-dimensional definition of participation used in the family of Participation-Related Constructs framework (fPRC) (Imms et al., 2017). Within this framework, attendance is seen as a primary prerequisite for participation. A second key element is involvement, as in experiencing engagement, persistence, and affect while doing an activity. Participation in the fPRC is related to both internal and external factors. Internal factors are individual activity competence (skills required to perform the activity), sense of self (such as self-efficacy), and preferences. External factors concern the environment, which exists independently of the individual and the context (the actual interaction between the individual and the environment). This framework can be used to describe relationships between personal and environmental factors that are vital for the participation of persons with or without disabilities in PE. All participation in PE occurs in a context (Imms et al., 2017), and experienced participation depends on prerequisites for participation, i.e., what a person can do, wants to do, has the opportunity to do, and is not prevented from doing (Mallinson and Hammel, 2010). For optimal involvement, the activity needs to be perceived as meaningful (Kang et al., 2014). Functioning in complex PE activities also requires physical, social, affective, and cognitive functional skills (Bailey et al., 2009). In this study, *activity competence* is operationalized as self-reported perceived physical and socio-cognitive functional skills. *Sense of self* as PE-specific and general school self-efficacy and prerequisites for participation (named aptitude to participate) in PE is operationalized as *preferences*.

### Self-Perceived Functioning

Students with disabilities are over-represented in groups at risk of failing PE in Sweden (Bråkenhielm, 2008). Special educational research consistently reports, e.g., Pijl et al. (2008), that students in need of special support are less popular, have fewer friends, and are less often members of subgroups. However, self-ratings show that they do have positive perceptions of self-concepts, social acceptance, and academic achievement and their ratings do not differ from their typically functioning peers (Avramidis, 2013). Peer relations may be fewer but seem to be more important to their academic self-concept (Allodi, 2000). Transitioning into secondary school in Sweden means higher educational demands in a criterion-referenced grading system, i.e., to what degree intended learning outcomes are acquired (The Swedish National Agency for Education, 2018). Explicit academic demands may affect student general school self-efficacy, i.e., academic, social, and emotional self-efficacy (Muris, 2001). Adolescents compare physical performance with each other, rather than in relation to a fixed set of grading criteria, which affects student’s motivation to participate (Jacobs et al., 2002). High self-efficacy promotes students’ participation, motivation, and expectation of future success (Chase, 2001). Amotivation may be caused by personal factors, such as students’ low outcome expectancy, low effort, and low capacity beliefs, i.e., PE-specific self-efficacy. Physical self-concepts, encompassing physical functioning, were positively associated with mastery and performance approach goals, as opposed to skill-related goals based on comparisons between peers (Hagger et al., 2011). Functioning in PE is probably related both to perceptions of action (perceived physical functional skills) and to perceptions of interaction (perceived socio-cognitive functional skills).

### Adolescent Developmental Processes in a PE Context

As students grow older, student self-efficacy seems to drop during secondary school, despite increased knowledge with age. This might be due to multiple factors, for example, early adolescence is a time where biological, environmental/organizational, and social–cognitive transitions concur (Vaz, 2010). Adapting to a new environment with higher demands is an example of an organizational transition. Adding to the stress of changes associated with school transition, reduced self-efficacy in early adolescence can be explained by environmental factors (Schunk and Pajares, 2010). For example, in secondary school, competition may be more emphasized, and teacher–student social interactions with feedback on student progress become less frequent. There is also a social–cognitive transition, which is connected to a person’s functioning, development, and identity (Vaz, 2010). Competence and capacity is commonly appraised in relation to peers, and these personal self-evaluative factors affect the adolescents’ motivation to participate in learning (Jacobs et al., 2002). Schunk and Pajares (2010) propose that self-efficacy beliefs in students tend to decline as they advance through school. However, the expanded social reference group, i.e., peers, and higher educational demands require students to reassess their abilities. Self-assessment accuracy improves as students gain task experience and engage in social comparisons, which in turn stabilizes the correspondence between self-efficacy and performance. Providing students with instructions, opportunities to practice self-evaluation, and feedback on their progress can further calibrate this correspondence. According to Jacobs et al. (2002), the decline in self-competence beliefs in sports accelerate during secondary school years. Students inevitably compare their performances in a competitive environment, such as a PE context, where traditional views of “a fit body” are fostered (Fitzgerald, 2005). Due to puberty onset, biological differences between adolescents at this stage are obvious, especially if students have physical disabilities. In Sweden, the entrance to adolescence can be said to take place during the last 3 years of compulsory school, i.e., school years 7–9 (ages 12.5–16.5) referred to as secondary school. Therefore, longitudinal approaches are needed to study the directionality of relationships and stability of scores and provide insight to developmental trends (Sabiston et al., 2014) of students’ self-reported experiences in early adolescence. Moreover, students with disabilities need to be studied as a separate group (Bertills et al., 2018a).

### Rationale, Aim, and Research Questions

The objectives of this study are to explore the development of participation-related constructs (Imms et al., 2017) as perceived and self-reported by students. The aim is twofold, to explore how processes of students’ perceived PE-specific self-efficacy, general school self-efficacy, and aptitude to participate in PE develop over time. Furthermore, the aim is to investigate how these processes are related to perceived physical and socio-cognitive functional skills and achievement (grades in PE). Groups of students with disabilities, high grades (A–C), and low grades (D–F) in PE from year 6 (before transitioning into secondary school) are compared. Research questions are as follows:

1. What changes in self-perceptions of self-efficacy (PE-specific and general) and aptitude to participate between year 7 and 9 are there in the three target groups?
2. How do relationships between perceived functional skills (socio-cognitive and physical), self-efficacy (PE-specific and general), and aptitude to participate change over time in the three target groups?
3. What are the relationships of developmental processes of students’ perceived self-efficacy (PE-specific and general) and aptitude to participate in PE with perceived functional skills and achievement (grades in PE)?

## Materials and Methods

A longitudinal study design was used with student questionnaires distributed and data collected from 25 classes at two timepoints. This study is part of a larger study (*n* = 450) and based on two separate waves of data collection from Swedish mainstream inclusive secondary school years 7 and 9 in a region of the south of Sweden. Presented in the current study are analyses of student self-reports collected at timepoint 1 (T1), year 7 and timepoint 2 (T2), year 9.

### Participants

Students with disabilities were specifically targeted and recruited first. All their classmates were approached thereafter. Classmates who enrolled were assigned to a group of students with either high (A–C) or low grades (D–F) in PE, depending on their grade in PE the previous year (see Table 1). For this study, purpose questionnaire data was analyzed from the 105 students in the target groups (23% of the total sample), who participated at both data collections. The group of students with disabilities was diagnosed with physical (*n* = 11, 7 boys), neuro-developmental (*n* = 12, 9 boys), intellectual (*n* = 2 boys), or a combination of two or more (*n* = 3, 2 boys) disabilities.

**TABLE 1:** Student participants.

Data	N	M	F	Disability	M	F	A–C	M	F	D–F	M	F
T1	121	55	45	30	73	27	55	40	60	36	64	36
T2	105	55	45	28	71	29	47	42	58	30	60	40

*M = percent of male participants, F = percent of female participants.*

### Data Collection Instruments

Student questionnaires (see Supplementary Material) were evaluated in a trial study (Bertills et al., 2018a). Internal consistency, factor structure, and relations between measures were investigated. Reliability and validity was analyzed with the first wave of data collection, and the questionnaires were deemed to adequately measure students’ perceived self-efficacy (PE-specific and general school self-efficacy), aptitude to participate in PE, and perceived physical and socio-cognitive functional skills. A simplified version was created to make the questionnaires accessible to all and used when needed to include students with mild intellectual disability in regular inclusive schooling. In this version, wording was simplified, the amount of text was reduced, and responses were colored in the PE-specific self-efficacy instrument (shades of orange to green). Assistants supplied with the original version aided these students.

#### Achievement

The Swedish national grading system is designed to measure knowledge and skills against a fixed set of predetermined criteria on an A–F grading scale. Students are assessed depending on the degree to which the student reaches the intended learning outcomes, A = very well, C = relatively well, E = partly, and F = fail. Grades B and D are rewarded when a student has met a considerable share of knowledge requirements for grade A and grade E, respectively.

#### Student Questionnaires

##### PE-specific self-efficacy

Task-specific items matching the PE syllabus with activities commonly occurring in PE lessons were developed according to recommendations concerning how to measure domain specific self-efficacy (Bandura, 2006). The scale created for this study purpose showed strong internal consistency, α = 0.93, at both timepoints. The items covered the three core content syllabus components: movement (gymnastics, team sports, etc., eight items), health and lifestyle (e.g., plan, do, evaluate your own exercise program targeting individually set goals, seven items), and outdoor life and activities (e.g., orienteering, five items). Students responded to exclamations initiated by *report how you perceive your knowledge and skills to…* on a scale ranging from 1 to 6, 1 = not at all good to 6 = very well, corresponding to grades A–F (mean T1 = 4.53, T2 = 4.69). The scale was dichotomized into low = 1–3 and high = 4–6.

##### Aptitude to participate in PE

Vital components for wanting to participate in PE (student preferences) were measured, e.g., opportunity and ability to participate, safety, support, and grading awareness (seven items). Strong internal consistency, α = 0.81, was seen at both timepoints. Scale range and dichotomization were similar to the instrument PE-specific self-efficacy (mean T1 = 5.02, T2 = 5.04).

##### Perceived functioning

Focusing on students with disabilities, i.e., with restricted functioning as diagnosed by a doctor, requires measurements of self-perceived functioning. The original proxy-rated index of children’s functional abilities (Simeonsson and Bailey, 1991) was adapted into a self-rating instrument (Bertills et al., 2018a). Physical functional skills refer to experienced functioning in the hands, arms, and legs. Moreover, socio-cognitive functional skills refer to experienced elements of general health, communicative, social, behavioral, and problem-solving skills. Perceived skills, relative to peers, were self-rated on a scale of 1–6, indicating 1 = profound difficulties to 6 = no difficulties. Each individual received a total sum, and items were dichotomized into physical functioning (0–7, low = 0–6, typical = 7) and socio-cognitive functioning (0–20, low = 0–14, typical = 15–20). In a total sample where typical functioning is perceived by a majority of the students, typical physical functioning was perceived by 76% of the students at T1 (α = 0.77) and 83% at T2 (α = 0.83), and socio-cognitive functioning by 72% at T1 (α = 0.74) and 76% at T2 (α = 0.75).

##### General school self-efficacy

A collective measurement of a general sense of school self-efficacy in adolescents was used. The translated instrument showed similar internal consistency as the original version of α = 0.88 (Muris, 2001). The scale initiated by *how well do you perceive that you can/succeed in* included 24 items with eight items each measuring aspects of academic, social, and emotional skills ranging from 1 to 5, 1 = *not at all* to 5 = *very well* (mean T1 = 3.71, T2 = 3.66). Dichotomization was based on the mean.

### Procedures

Data collection procedures were elaborated from the trial study to assist students in need of special support, e.g., placement, further explanations, reading aloud, and adult assistance. The simplified version was used when needed, so that all students could complete the questionnaire. Assistants supplied with the original version aided these students. Questionnaires were distributed to students in their home classrooms or adjacent group room and collected by the researchers approximately one term after transition into secondary school (year 7 = T1) and repeated the last term before graduating (year 9 = T2). Owing to circumstances, the researcher completed the questionnaire in telephone communication with two students with disabilities at T2.

### Statistical Analysis

To explore student self-efficacy (PE-specific and general) and aptitude to participate, mean scores were calculated individually for each scale, excluding cases with more than 25% missing values. Analysis of variance (ANOVA) (Bird, 2004) was used to investigate mean differences between average scores in the groups of students with disabilities, high grades (A–C), and low grades (D–F). Paired-sampled *t*-tests were conducted to detect change over time within the scales. Spearman’s rho was used to examine correlations (Field, 2013) of perceived self-efficacy (PE-specific and general) and aptitude to participate with physical and socio-cognitive functioning skills and achievement in the study sample and in each group separately. Correlations less than 0.30 were considered as weak, and more than 0.60 as strong. Scale correlations were then compared between T1 and T2 using Fisher’s *r* to Z transformations (Siegel and Castellan, 1988) to detect differences between the three student groups. The potential of physical and socio-cognitive functional skills to predict elevated PE-specific self-efficacy, aptitude to participate in PE, and general school self-efficacy was examined. A series of binary logistic regression analyses was performed, using physical and socio-cognitive skills as predictors.

### Ethics

All students actively consented to participate in the current study. Written informed consent was obtained from the participants and their parents. The study was approved by the Ethical Review Board, Linköping, Sweden (2013/508-31).

## Results

### Perceived PE-Specific Self-Efficacy, Aptitude to Participate, and General School Self-Efficacy Over Time

Averages of self-report data from the scales remained relatively stable over time (see Table 2). Students with disability reported lower means and larger within group variance than the other groups. Comparisons between timepoint 1 (T1), year 7 and timepoint 2 (T2), year 9 showed significantly elevated PE-specific self-efficacy, mainly due to the significant increase seen in students with low grades.

**TABLE 2:** Descriptive scales in the study sample and in each group separately.

		Sample (*n* = 105)		Disability (*n* = 28)		A–C (*n* = 47)		D–F (*n* = 30)	
		T1	T2	T1	T2	T1	T2	T1	T2
PE-specific self-efficacy	Mean	4.53*	4.68*	4.19	4.22	4.81	4.95	4.40**	4.70**
	SD	0.84	0.86	1.05	1.14	0.59	0.64	0.83	0.68
Main effect		*t* = 2.004						*t* = 3.064	
		*p* = 0.048						*p* = 0.005	
Aptitude to participate	Mean	5.02	5.04	4.71	4.60	5.23	5.28	4.98	5.10
	SD	0.86	0.90	1.01	1.23	0.67	0.65	0.89	0.76
General self-efficacy	Mean	3.71	3.66	3.54	3.63	3.83*	3.70*	3.69	3.64
	SD	0.51	0.55	0.51	0.68	0.44	0.46	0.58	0.55
Main effect						*t* = −2.135			
						*p* = 0.038			
Physical function	Mean	6.38	6.49	5.86	5.79	6.68	6.77	6.40	6.70
	SD	1.41	1.39	1.84	2.10	0.89	0.94	1.52	0.92
Socio-cognitive function	Mean	16.05	16.21	14.64	15.21	16.89	17.02	16.03	15.90
	SD	3.41	3.23	3.84	3.51	3.16	2.92	3.00	3.19

**p < 0.01, *p < 0.05. Significant within group difference. PE-specific self-efficacy (ranging 1–5), aptitude to participate (ranging 1–6), general school self-efficacy (ranging 1–6), physical functional skills (ranging 0–7), socio-cognitive functional skills (ranging 0–20).

Students with disabilities reported higher general school self-efficacy (non-significant), less aptitude to participate, and consistent lower perceived physical functional skills than students with either high (A–C group) or low grades (D–F group). Differences increased between students with disabilities and the A–C group and were significant at both timepoints in all scales but two. Socio-cognitive skills were non-significant at T2, and general school self-efficacy did not show significant difference at either timepoint. In the A–C group, there was a significant decline in general school self-efficacy over time (*t* = −2.14, *p* = 0.038).

#### Interrelationships of Self-Efficacy (PE-Specific and General) and Aptitude to Participate

Partial correlations were investigated between the scales self-efficacy (PE-specific and general) and aptitude to participate, controlling for the third variable at each timepoint (see Table 3). Due to interrelationships between the scale, e.g., PE-specific self-efficacy was strongly related to the instrument *aptitude to participate* in PE (T1 *r* = 0.764, T2 *r* = 0.752, *p* < 0.01), the estimated relations would probably be influenced by each other.

**TABLE 3:** Partial correlations between self-efficacy (PE-specific and general) and aptitude to participate at T1 and T2, controlling for the third variable.

	Timepoint 1 (year 7)		Timepoint 2 (year 9)	
	PE-specific self-efficacy	Aptitude to participate	PE-specific self-efficacy	Aptitude to participate
**Study sample (*n* = 102)**				
Aptitude to participate	0.625**		0.666**	
General self-efficacy	0.413**	0.151	0.271*	0.173
**Disabilities (*n* = 25)**				
Aptitude to participate	0.685**		0.717**	
General self-efficacy	0.212	0.292	0.113	0.391*
**A–C (*n* = 44)**				
Aptitude to participate	0.643**		0.477**	
General self-efficacy	0.204	0.280	0.327*	0.173
**D–F (*n* = 27)**				
Aptitude to participate	0.546**		0.591**	
General self-efficacy	0.737**	−0.119	0.436*	−0.032

**p < 0.01, *p < 0.05.

The relationship between the different scales was relatively stable within the student groups over the two timepoints. Comparing groups, association differences of scales were non-significant. Students with disabilities showed weaker non-significant association between PE-specific self-efficacy and general school self-efficacy at T2 than the other two groups. Using Fisher’s *r* to Z transformations, this association was found to be significantly stronger at T1 in the D–F group than in the other groups [disabilities (Z = 2.47, *p* = 0.014), A–C group (Z = 2.87, *p* = 0.004)]. Students’ perceived PE-specific self-efficacy and aptitude to participate were strongly associated at both timepoints. This association became stronger over time in students with disabilities and in the D–F group than in the A–C group, who reported a weaker association at T2.

Relationships within self-reported PE-specific self-efficacy were stable across the groups (students with disabilities *r* = 0.40, *p* = 0.035; the A–C group *r* = 0.53, *p* < 0.01; and the D–F group *r* = 0.76, *p* < 0.01). A significant difference in the stability of PE-specific self-efficacy over time was found between students with disabilities and students in the D–F group (Z = 2.09, *p* = 0.037).

### Perceived Physical and Socio-Cognitive Functioning Skills in Relation to Self-Efficacy (PE-Specific and General) and Aptitude to Participate in PE

For students with disabilities, associations between perceived physical functional skills and all other measures changed from being weak at T1 to explaining 31–47% of the variance at T2 (see Table 4). Initial self-reports of physical functional skills remained stable over time in this group (*r* = 0.577, *p* < 0.01). Physical functional skills at T2 in the D–F group were close to zero related to their aptitude to participate, and associations with the other measures were relatively small at either timepoint. Physical functional skills had little or no relevance for any of the measures in the A–C group at either timepoint.

**TABLE 4:** Correlations over time between students’ perceived functional skills (physical and socio-cognitive), self-efficacy (PE-specific and general), and aptitude to participate in the study sample and in each group separately.

	PE-specific self-efficacy		Aptitude to participate		General self-efficacy	
	T1	T2	T1	T2	T1	T2
**Physical skills**						
Study sample (*n* = 105)	0.105	0.266**	0.093	0.212*	0.064	0.307**
Disabilities (*n* = 28)	0.079	0.554**	0.084	0.562**	−0.003	0.687**
A–C (*n* = 47)	0.038	−0.059	−0.104	−0.067	−0.155	−0.035
D–F (*n* = 30)	0.050	0.164	0.209	−0.002	0.180	0.205
**Socio-cognitive skills**						
Study sample	0.521**	0.398**	0.435**	0.260**	0.653**	0.466**
Disabilities	0.330	0.497**	0.518**	0.407**	0.609**	0.411**
A–C	0.426**	0.239	0.208	0.078	0.430**	0.402**
D–F	0.673**	0.346	0.499**	0.186	0.833**	0.635**

**p < 0.01, *p < 0.05.

Regarding perceived socio-cognitive skills, overall moderate significant associations with all scales were observed in students with disabilities at both timepoints (see Table 4). Although associations generally declined in all groups, students with disabilities were found to have stronger association with PE-specific self-efficacy over time. General school self-efficacy was relatively strongly associated with perceived socio-cognitive skills in all groups at both timepoints. The most affected by socio-cognitive functional skills at T1 was the general school self-efficacy of students in the D–F group (explaining 70% of the variance). Although this impact declined over time, 40% of the variance at T2 was still explained in the D–F group.

Differences of physical functional skills between students with disabilities and the other two groups were significant (see Table 5). At T2, students in the study sample who reported better physical functional skills were also five times more likely to report high general school self-efficacy at T2 (see Table 6).

**TABLE 5:** Fisher’s *r* to Z estimations of group differences at T2 between physical skills and PE-specific self-efficacy (SEinPE), aptitude to participate, and general school self-efficacy (GeneralSE).

	SEinPE			Aptitude to participate			GeneralSE		
Group	Z	*R*	*p*	Z	*r*	*P*	Z	*r*	*p*
Disability	0.554			0.562			0.687		
A–C	−0.059	2.73	0.006	−0.067	2.81	0.005	−0.035	3.50	0.000
D–F	0.164	1.65	0.098	−0.002	2.3	0.022	0.205	2.28	0.022

**TABLE 6:** Binary logistic regression models in the study sample (*n* = 105) with elevated PE-specific self-efficacy, aptitude to participate, and general school self-efficacy as outcome variables and with socio-cognitive and physical skills as independent variables.

	Elevated PE-specific self-efficacy				Elevated aptitude to participate				Elevated general school self-efficacy			
	T1		T2		T1		T2		T1		T2	
	OR (95% CI)	*R*^2^_N_	OR (95% CI)	*R*^2^_N_	OR (95% CI)	*R*^2^_N_	OR (95% CI)	*R*^2^_N_	OR (95% CI)	*R*^2^_N_	OR (95% CI)	*R*^2^_N_
Elevated socio-cognitive skills	2.647	0.057*	3.881	0.094*	4.664	0.143**	2.086	0.032	9.583	0.232**	4.103	0.096*
	(1.053–6.656)		(1.355–11.114)		(1.873–11.611)		(0.838–5.194)		(3.030–30.307)		(1.401–12.016)	
Elevated physical skills											4.886	0.088*
											(1.320–18.090)	

*R*^2^_*N*_ = Nagelkerke’s pseudo *R*^2^. **p < 0.01, *p < 0.05.

Significant effect of perceived socio-cognitive skills on PE-specific self-efficacy had over time increased in the study sample (see Table 7). One significant model was found, where students in the study sample with initial high PE-specific self-efficacy were nine times more likely to report high PE-specific self-efficacy at T2, explaining 15.4% of the variance (Nagelkerke *R*^2^). Adding improved socio-cognitive skills (mean value of change) into this model further raised the likelihood of high PE-specific self-efficacy at T2 by three times, explaining 24.4% of the variance (Nagelkerke *R*^2^).

**TABLE 7:** Summary of logistic regression analysis for perceived functional socio-cognitive change (mean value) predicting PE-specific self-efficacy at T2 in the study sample (*n* = 105).

Variable	Model 1						Model 2					
	B	SE B	Wald χ^2^	*p*	OR	95% CI OR	B	SE B	Wald χ^2^	*p*	OR	95% CI OR
PE-specific self-efficacy	1.72	0.54	10.22	0.001	5.581	[1.94, 16.01]	2.2	0.61	13.01	0.000	9.114	[2.74, 30.28]
Socio-cognitive skills							1.15	0.47	6.07	0.014	3.172	[1.94, 16.01]

### Perceived Self-Efficacy (PE-Specific and General), Aptitude to Participate in PE, and Functioning in Relations to Achievement

Relationships between PE achievement and students’ perceived self-efficacy (PE-specific and general), aptitude to participate in PE, and perceived functional skills (physical and socio-cognitive) were investigated (see Table 8). Findings showed that PE grades year 7 explained 61% of the variance in the final PE grades of students with disabilities, compared with the A–C group (28%) and the D–F group (40%). In the group of students with disabilities, PE grades were moderately associated with PE-specific self-efficacy (*r* = 0.461, *p* < 0.05) and aptitude to participate at T1 (*r* = 0.504, *p* < 0.01) and strongly at T2 (*r* = 0.679/*r* = 0.739, *p* < 0.01). Compared with their peers, the A–C group showed weak associations at both timepoints, and the D–F group showed weak associations at T1 and strong at T2. A significant association between PE grade at T1, year 7 and perceived socio-cognitive skills at T2, year 9 was found in the D–F group (*r* = 0.519, *p* < 0.01).

**TABLE 8:** Correlations over time of achievement in PE with students’ perceived self-efficacy (PE-specific and general), aptitude to participate, and functioning (physical and socio-cognitive).

Achievement	SEinPE	SEinPE	Apt.part	Apt.part	GSE	GSE	Phys	Phys	Socio-cog	Socio-cog	Final grade
	T1	T2	T1	T2	T1	T2	T1	T2	T1	T2	
**Study sample**											
Year7	0.451**	0.390**	0.387**	0.483**	0.235*	0.214*	0.142	0.154	0.249*	0.365**	0.732**
Final	0.471**	0.524**	0.408**	0.585**	0.281**	0.141	0.181	0.236*	0.274**	0.274**	
**Disability**											
Year7	0.461*	0.492**	0.504**	0.565**	0.167	0.283	0.044	0.203	0.038	0.173	0.783**
Final	0.656**	0.679**	0.632**	0.739**	0.338	0.311	−0.001	0.291	0.085	0.367	
**A–C**											
Year7	0.362*	0.126	0.171	0.282	0.151	0.136	0.222	0.024	0.235	−0.001	0.532**
Final	0.209	0.261	0.072	0.392**	0.120	0.030	0.297*	0.230	0.188	0.057	
**D–F**											
Year7	0.340	0.389*	0.332	0.508**	0.177	0.270	−0.073	0.003	0.033	0.519**	0.635**
Final	0.501**	0.586**	0.511**	0.622**	0.349	0.179	0.003	−0.064	0.171	0.113	

Achievement = spring grade in PE year 7 (Year7) and final grade in PE year 9 (Final), SEinPE = PE-specific self-efficacy, Apt.part = aptitude to participate, GSE = general school self-efficacy, Phys = physical functional skills, Socio-cogn. = socio-cognitive functional skills, timepoint 1, year 7 (T1) and timepoint 2, year 9 (T2); **p < 0.01, *p < 0.05.

## Discussion

### Developmental Processes of PE-Specific Self-Efficacy, Aptitude to Participate, and General School Self-Efficacy

Students with disabilities rate themselves as having lower levels of aptitude to participate and physical functional skills. Relationships between PE-specific self-efficacy and aptitude to participate at both timepoints were relatively strong in all groups. These associations became stronger over time in the group of students with disabilities and the group with low grades. These findings are in line with those of Ntoumanis et al. (2004) regarding causes of amotivation. This group demonstrated that capacity beliefs, i.e., PE-specific self-efficacy, over time become strongly related to whether the groups of students with disabilities and those with low grades were motivated to participate in PE or not.

The finding that PE-specific self-efficacy increased in secondary school, for all groups, is promising. Especially since this contradicts findings that self-competence beliefs in sports decline in secondary school (Jacobs et al., 2002). During the last two decades, PE in Sweden has been increasingly focused on health-directed learning (Lundvall and Brun Sundblad, 2017). The PE-specific questionnaire was constructed in accordance with the Swedish syllabus on a scale corresponding to the A–F grading scale. A report by The Swedish Schools Inspectorate (2018) summarizes that PE is a popular school subject among adolescents in secondary school, but the gap between active and inactive adolescents is increasing. Low grades in PE, occurring in the group of students with low grades but also partly in the group of students with disabilities, are likely associated with less involvement in extracurricular physical activities. In this study, a significant increase of PE-specific self-efficacy was found in the group of students with low grades as compared with the groups of student with disabilities and those with high grades. Self-ratings of PE-specific self-efficacy for students with low grades were consistent and highly correlated between T1 and T2. In addition, aptitude to participate remained high over time in this group. Combined, these findings imply that the students who benefit the most from school-based PE are those with low grades. The significantly stronger association with general school self-efficacy found in this group of students at T1, combined with significantly stable ratings and increased PE-specific self-efficacy, further implies that acquired knowledge and skills in PE may compensate for the loss of general school self-efficacy seen in students with low grades.

For students with disabilities, another trend could be discerned. In the present study, this group reported higher general school self-efficacy after 3 years of secondary school (see Table 2). This result was non-significant but contradicts what was found in the other two groups and also previous findings that show an overall decline of general school self-efficacy in adolescence (Schunk and Pajares, 2010). Maturity pace, environmental factors, and acceptance may explain the relatively unstable ratings of general school self-efficacy over time in the group of students with disabilities. General school self-efficacy in secondary school may be affected by students with disabilities being more sensitive to environmental factors, such as classroom climate (Bertills et al., 2018b). It is also possible that they develop at a slower pace than their typically functioning peers (Lygnegård et al., 2018). Additionally, acceptance of “being different” by themselves and others, potentially aided by school-based support services, may have contributed to the positive trend of general school self-efficacy. A previous cross-sectional study showed that when PE teachers rated their teaching skills high, students with disabilities reported lower self-efficacy (PE-specific and general) and aptitude to participate in the initial phase of secondary school. In contrast, their peers rated higher self-efficacy (PE-specific and general) and aptitude to participate (Bertills et al., 2018b). One reason for this might be the higher stakes’ environment at the transition to secondary school. For a person with lower perceived physical functional skills, grading standards may be set too high to be perceived as achievable. The environment may also hinder the full participation, i.e., attendance and involvement (Imms et al., 2017), of students who do not fulfill traditional views of “a fit body” (Fitzgerald, 2005). The strong association between PE grade year 7 and final PE grade year 9 indicates that students with disabilities are “stuck” in the Swedish criterion referenced grading system. This finding contradicts with the ideal of inclusive, equitable schooling (Nilholm, 2006; Ainscow, 2012). Adding further to our suggestion from previous findings (Bertills et al., 2018b), knowledge and skills of students in need of special support in PE ought not to be assessed according to the fixed set of predetermined criteria seen in the Swedish grading system. Grading criteria need to be adapted and communicated in a way that makes students with disabilities understand that it is ok to do PE activities differently. However, our results also imply that school-based PE provides disadvantaged students with participatory gains. Findings show that PE grades and PE-specific self-efficacy and aptitude to participate are related, and that the strongest associations were found in the group of students with disabilities. Over time these associations became stronger in the group of students with disabilities and in the group of students with low grades. The importance of creating meaningful learning experiences that students with disabilities will want to participate in Haegele and Sutherland (2015) must be emphasized. Our study also suggests that promoting PE-specific self-efficacy is yet another aspect to consider. Participation boosts PE-specific self-efficacy, and capacity beliefs boost the desire to join in.

### Perceived Functioning in Relation to Self-Efficacy (PE-Specific and General) and Aptitude to Participate in PE

How functioning is perceived to affect performance in PE might change over time. Developmental changes in adolescence occur at different times and paces. Little is known about school transitions for students disadvantaged by disability (Hughes et al., 2013) and the development of student functioning in PE over time. Perceived physical functional skills of students with disabilities seem to limit their aptitude to participate in PE. Ratings of functional restrictions of their physical skills (arms, legs, and hands) were stable over time in students with disabilities. However, moving from close to zero impact at T1 (see Table 4), self-rated physical functional skills became strongly related to self-efficacy (PE-specific and general) and aptitude to participate in PE at T2. This change only occurred in students with disabilities (see Table 5) and may be due to changes in frame of references for this group, from perceptions of effort as a reward to comparing their physical skills with other students (Jacobs et al., 2002). Previous reports on declining participation in PE in secondary school (Säfvenbom et al., 2014; The Swedish Schools Inspectorate, 2018) seem to be connected to students’ perceived functioning. In Sweden, it is legislated that special support should be provided if students risk failing one or more subjects (The Swedish National Agency for Education, 2018). Evidence for beneficial effects of physical activity interventions on overall academic performance is inconclusive (Singh et al., 2019). Ericsson and Karlsson (2014) provided evidence in a 9-year intervention study that adapted motor skills training during compulsory school years had positive effects not only on motor skills but also on final grades. However, special support in PE is rarely provided in secondary school. In a previous observational study, the primary source of support to students with disabilities seemed to consist of closer proximity to the teacher (Bertills et al., 2019). Higher student engagement was observed in the groups of students with disabilities and those with low grades in conditions where PE teachers offered whole group self-sustaining activities. The PE teachers could then compensate for the lack of assistance by allocating teacher support. Closer proximity was achieved in small group activities, where differentiated challenges and individualized support and feed-back/feed-forward could be provided. In the current study, our results show that students perceiving physical restrictions were considerably less likely to experience high general school self-efficacy in the final phase of mainstream inclusive secondary school. This finding highlights the importance of fostering a mastery climate emphasizing effort before physical skills (Valentini and Rudisill, 2004; Harwood et al., 2015).

Self-rated perceived socio-cognitive functional skills, i.e., general health, behavioral, social, communicative, and problem-solving skills, improved over time in the study sample (see Table 2). In line with previous research linking self-regulation to learning (Zimmerman, 2000), our findings show stronger associations over time between socio-cognitive functional skills and perceived PE-specific self-efficacy in the group of students with disabilities (Table 4) with the opposite found in the other two groups. Maybe the different patterns of associations imply that elevation of perceived socio-cognitive functional skills occurs at a different pace in each group, with a slower pace in students with disabilities. Consistency in ratings of socio-cognitive functional skills was found in students with high grades, which indicate that they are not as affected as their peers. This may signal early maturity, which supports previous research that initial high efficacy to self-regulate student actions leads the way to future success (Caprara et al., 2004). It is also likely that students with disabilities mature at a slower pace (Lygnegård et al., 2018). Another explanation is the previously mentioned change in frame of reference. At T1, students with disabilities responded negatively to teachers who worked systematically with grading (Bertills et al., 2018b), and stronger associations were identified between functional skills and PE-specific self-efficacy at T2. Partaking regularly in PE and practicing prosocial activities with set rules together with peers may enhance students’ ability to regulate socio-cognitive functional skills. Eventually, they would adapt to the new environment, which in turn positively would affect their PE-specific self-efficacy, and probably also their aptitude to participate in PE. A positive learning climate, where effort is promoted before competition, and where students are allowed to perform differently, may also boost self-efficacy, aptitude to participate, and perceived socio-cognitive functional skills. Schunk and Pajares (2010) further argue that as student self-evaluations become more accurate, discrepancies between self-efficacy and performance are leveled out. The group of students with low grades was the most affected by limitations in socio-cognitive functional skills at T1 (Table 4). Although improving during secondary school, skills, such as being able to communicate, behave, and solve problems, considerably affect the general school self-efficacy of this group of students. Our results confirm previous findings that initial high self-efficacy remains over time (Chase, 2001), but also that PE-specific self-efficacy is promoted by improved students’ socio-cognitive functioning (Table 7). The positive association in students with low grades between PE grade at T1 and perceived socio-cognitive functioning at T2 also has implications for how PE teachers should reason about grading. One common direction to take is to initially award a lower grade, for the students to improve toward the intended learning outcomes. Maybe it should be the other way around, as initial PE grades affect future socio-cognitive skills positively in the group of students with low grades. Belief in one’s capability may spread across domains and influence future choices (Pajares and Urdan, 2006). We therefore suggest that there seems to be overall gains to be found in supporting the development of students’ perceived socio-cognitive skills in a PE environment. This suggestion has implications for the allocation of resources. Empowering students’ socio-cognitive skills would probably have direct effects on self-efficacy and aptitude to participate in PE and presumably indirect effects on overall school and future life outcome.

## Limitations

Due to limited sized participant groups in this study, tendencies and patterns of developmental processes were explored, rather than significance. Results should be used with caution. Generally, correlations > 0.30 were taken into consideration. Asking students their perceived age of maturity would have added further nuances of student developmental processes. Two timepoint measures only show bidirectional correlations. Collecting data a third time would have better informed the discussion of maturity pace on how self-efficacy, aptitude to participate, and functioning develop differently in the three groups studied. Gender differences were controlled for at T1 in a sample of 450 students with no significant differences found. It was decided to focus on the target groups rather than gender. However, the fact that there were more boys in the groups of students with disabilities and those with low grades and more girls in the group with high grades may have affected results over time. Group dynamics changed in some of the classes investigated, due to a large number of refugees (not included in the current study) added between T1 and T2 that may indirectly have impacted the results. Students with disabilities are often excluded from partaking in research concerning their experiences (Alderson and Morrow, 2011), due to ethical considerations (Qi and Ha, 2012) and exclusive research methodology (Haegele and Hodge, 2017). To ensure successful completion by students in need of special support, accommodations were made, such as providing a separate room, a simplified version of the questionnaire, and reading aide (Bertills et al., 2018a).

## Conclusion

The aims were to explore how processes of students’ perceived PE-specific self-efficacy, general school self-efficacy, and aptitude to participate in PE develop over time, as well as how these processes were related to students’ perceived physical and socio-cognitive functional skills and achievement. Student PE-specific self-efficacy is strengthened over time, and associations between PE-specific self-efficacy and aptitude to participate are consistent. These findings imply that attendance in PE stimulates engagement and learning and confirms that learning takes place in accordance with the current Swedish PE syllabus. Consequently, PE-specific self-efficacy may be considered as a positive outcome of school-based PE and could be used to complement a summative grade. Grades remained consistent over time for students with disabilities, indicating that grading criteria need to be adapted to PE ability, in order to adequately show student progress. The experienced feeling of having physical restrictions accelerates in students with disabilities. School interventions should aim at limiting negative effects of such negative experiences. Providing adapted motor skills training is one feasible way; another is to adopt an inclusive approach to facilitate participation. Meaningful learning experiences for students with disabilities are created in conditions where inclusive approaches are provided. Students with disabilities are more sensitive to the classroom climate, and it is reasonable to assume that inclusive teaching practices promote participation and boost self-efficacy. Allocating resources to support students’ socio-cognitive skills would probably generate positive effects on the classroom climate as well as on student general school self-efficacy, that is, academic, social, and emotional self-competence beliefs.

## Data Availability Statement

The raw data supporting the conclusions of this article will be made available by the authors, without undue reservation.

## Ethics Statement

The studies involving human participants were reviewed and approved by The Ethical Review Board, Linköping (2013/508-31). Written informed consent to participate in this study was provided by the participants’ legal guardian/next of kin.

## Author Contributions

KB adapted the instruments, distributed the questionnaires, collected and analyzed the data, and wrote the first draft. All authors interpreted the results and contributed to the writing and revising process.

## Conflict of Interest

The authors declare that the research was conducted in the absence of any commercial or financial relationships that could be construed as a potential conflict of interest.
