# Supplementary material for: Student Self-Efficacy and Aptitude to Participate in Relation to Perceived Functioning and Achievement in Students in Secondary School With and Without Disabilities
Source: Front Psychol. 2021 May 6;12:607329. doi: 10.3389/fpsyg.2021.607329 (PMC8134687; doi:10.3389/fpsyg.2021.607329)
Supplement: Supplementary file 1 [file Data_Sheet_1.docx]

Supplementary material

Student questionnaires

*General self-efficacy*

The scale to measure general self-efficacy was translated into Swedish on the creator’s approval. Displayed is the original version, Self-Efficacy Questionnaire for Children (SEQ-C) with its subscales academic (items 1, 4, 7, 10, 13, 16, 19, 22) social (2, 6, 8, 11, 14, 17, 20, 23) and emotional self-efficacy (3, 5, 9, 12, 15, 18, 21, 24), ranging 1-5, 1=not at all, 5=very well (Muris, 2001).

1. How well can you get teachers to help you when you get stuck on schoolwork?
2. How well can you express your opinions when other classmates disagree with you?
3. How well do you succeed in cheering yourself up when an unpleasant event has happened?
4. How well can you study when there are other interesting things to do?
5. How well do you succeed in becoming calm again when you are very scared?
6. How well can you become friends with other children?
7. How well can you study a chapter for a test?
8. How well can you have a chat with an unfamiliar person?
9. How well can you prevent to become nervous?
10. How well do you succeed in finishing all your homework every day?
11. How well can you work in harmony with your classmates?
12. How well can you control your feelings?
13. How well can you pay attention during every class?
14. How well can you tell other children that they are doing something that you don’t like?
15. How well can you give yourself a pep-talk when you feel low?
16. How well do you succeed in understanding all subjects in school?
17. How well can you tell a funny event to a group of children?
18. How well can you tell a friend that you don’t feel well?
19. How well do you succeed in satisfying your parents with your schoolwork?
20. How well do you succeed in staying friends with other children?
21. How well do you succeed in suppressing unpleasant thoughts?
22. How well do you succeed in passing a test?
23. How well do you succeed in preventing quarrels with other children?
24. How well do you succeed in not worrying about things that might happen?

*PE specific self-efficacy*

PE specific self-efficacy was created by the author in accordance with the Swedish PE syllabus and its three core content areas movement, health and lifestyle, outdoor life and activities (subscales MovementSE, items 1-8, HealthSE, items 9-15, OutdoorSE, items 16-20). The items were based on commonly occurring activities in PE lessons. The scale (ranging 1-6, 1=not good at all, 6=very good) correspond to the grading scale (F-A). The original version is in Swedish.

*Report how you perceive your skills and abilities to…*

MovementSE

1. …participate in dance
2. …participate with pace and rhythm in fitness programs
3. …participate in games
4. …participate in athletics
5. …participate in obstacle courses
6. …participate in gymnastics
7. …participate in ball games
8. …swim in various types of strokes

HealthSE

1. …set up goals for my training and physical activity
2. …choose, plan, carry out and evaluate my training and physical activity
3. …talk about my experience of activity and effects on health and physical capacity
4. …prevent injuries associated with games and sports
5. …describe risks associated with physical activity
6. …handle emergencies
7. …reason about how activities together with dietary and other factors can influence health and physical capacity

OutdoorSE

1. …plan, organize and carry out outdoor life activities
2. …act according to the rules of public access to land
3. …adapt clothing to weather conditions
4. …handle water emergencies
5. …orient myself in unfamiliar environments using maps and other aides

*Aptitude to Participate in PE*

The scale aptitude to participate in PE was created to capture motivational aspects to participate and perform in PE, items 2,5,6,7 (The Swedish Schools Inspectorate, 2011), and prerequisites for engagement in PE, items 1,3,4 (Maxwell et al., 2012). The scale (ranging 1-6, 1= disagree, 6=fully agree) correspond to the grading scale (F-A). The original version is in Swedish.

*Report how you perceive different activities in PE*

*I perceive that I…*

1. …usually CAN participate in PE
2. …usually “do my best” in PE
3. …feel safe in the changing room
4. …CAN participate in competitive games
5. …know what is expected from me in PE (knowledge and skills)

1. …actively participate in a sports club
2. …my teacher in PE encourages me

*Functioning*

A Swedish version (Roll-Pettersson, 2001) of the Abilities Index (Bailey, Simeonsson, Buysse, & Smith, 1993) was converted from being a proxy-rated into a self-report instrument of perceived functioning (ranging 1-6, 1=severely restricted function, 6=typical function). The scale was indexed into physical, items 1-3, and socio-cognitive, items 4-9, functional skills.

Physical functional skills

1. Hands
2. Arms
3. Legs

Socio-cognitive functional skills

1. General Health
2. Social skills
3. Behavioral skills
4. Communicational skills, understand others
5. Communicational skills, make myself understood
6. Cognitive skills (thinking and reasoning)
